# Regulation of DNA methylation in lesional tissue of children with atopic dermatitis

**DOI:** 10.3389/fmed.2025.1531777

**Published:** 2025-03-03

**Authors:** Demet Kartal, Muhammed Hanefi Dirican, Serpil Taheri, Mehmet Memiş, Eda Öksüm Solak, Salih Levent Cinar, Murat Borlu

**Affiliations:** ^1^Dermatology and Venereology Department, Erciyes University Medical School, Kayseri, Türkiye; ^2^Dermatology and Venereology Department, Tokat City Hospital, Tokat, Türkiye; ^3^Betul Ziya Eren Genome and Stem Cell (GENKÖK) Center, Erciyes University, Kayseri, Türkiye; ^4^Technology Transfer Office Application and Research Center, Bayburt University, Bayburt, Türkiye

**Keywords:** atopic dermatitis, DNA methylation, TET1-2 and DNMT gene expression, epigenetic, pediatric atopic dermatitis

## Abstract

**Background:**

Genetic and epigenetic mechanisms have been shown to play a role in the pathogenesis of atopic dermatitis (AD). However, the role of genes involved in the establishment of DNA methylation has not yet been demonstrated.

**Methods:**

A total of 15 pediatric patients with AD and 15 healthy volunteers were included in this study. The mRNA gene expression levels of eight different genes involved in the regulation of DNA methylation were examined in the blood and tissue samples.

**Results:**

The mRNA expression levels of *DNMT3A* genes were significantly increased, while the mRNA expression levels of *DNMT3B*, *TET1*, and *TET2* genes were statistically significantly reduced in the lesional tissue of patients compared to the control group. It was observed that the mRNA expression levels of *DNMT1, DNMT3A*, and *TET3* genes were increased, while the mRNA expression levels of *DNMT3L* and *TET1* genes were found to be decreased in the blood of the patients.

**Conclusion:**

The results indicated that the DNA methylation pattern in the patients was hypermethylated, especially in the lesional tissue. The data obtained may contribute to the understanding of the epigenetic regulation of AD and aid in the development of new diagnostic and treatment options.

## Graphic abstract

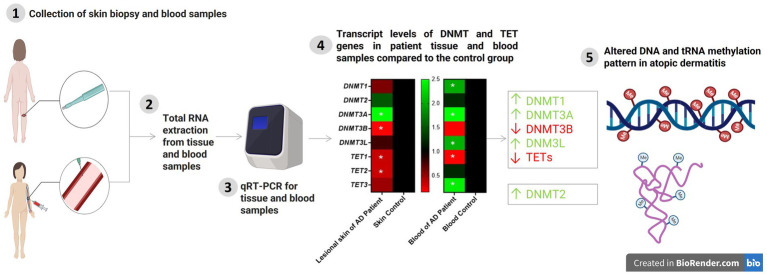

## Introduction

Atopic dermatitis (AD), also known as atopic eczema, is a common inflammatory skin condition, and it affects 15–20% of children and 7–10% of adults in developed countries (1, 2). AD is typically developed during childhood and is characterized by intensely pruritic, recurrent eczematous lesions (1). Patients with AD also have an increased risk of developing allergic rhinitis, food allergies, and asthma, making AD the first step in the atopic march (3).

AD is a multifactorial disease, and its pathophysiology is not yet fully understood. However, existing evidence suggest that AD is associated with impaired epidermal barrier function, activation of different immune cell types, and changes in the skin microbiome due to genetic and environmental factors. These mechanisms are thought to contribute to the clinical presentation of the disease (4). A correlation between AD and over 70 different genes has been demonstrated in previous studies (5). However, these genetic correlations only account for a small portion of the disease’s variability. Mutations in these genes have also been detected in healthy individuals. Familial inheritance and the increasing frequency of AD indicate that the disease may be an acquired epigenetic regulation disorder. Although epigenetic mechanisms are significant risk factors that play a role in familial allergic diseases, there are only a few studies on this topic in the literature (5).

Epigenetics is the study of heritable changes in gene expression without changes in the DNA sequence and is influenced by environmental factors. Key mechanisms of epigenetics include CpG methylation, histone modifications, and regulatory non-coding RNAs (ncRNAs). The deregulation of these epigenetic mechanisms has been associated with various human diseases. In dermatology, skin disorders, such as AD and psoriasis, significantly affect skin integrity and disrupt epidermal differentiation. It has been shown that the epigenetic mechanisms play a role in the disruption of epidermal differentiation. Recent studies have demonstrated that the dysregulation of epigenetic mechanisms occur in many inflammatory skin diseases, such as psoriasis (6). In particular, it has been shown that this disruption occurs locally in the skin tissue. The dysregulation of multiple epigenetic mechanisms, including abnormal DNA methylation, changes in histone modifications, and miRNA expression, has been shown to play a crucial role in the pathogenic processes of these skin conditions (7). Notably, differences in epigenetic signatures have been observed not only between the skin of healthy controls and the lesional skin of psoriatic patients but also between the lesional, perilesional, and unaffected skin of individual patients (8).

DNA methylation within these epigenetic mechanisms may occur in the promoter, exon, or intergenic regions of a gene. In the literature, studies on AD have examined the methylation profiles of numerous genes, demonstrating that these profiles in patients with AD differ from those in healthy individuals. However, changes in the methylation patterns of various genes, similar to mutations in several genes linked to Alzheimer’s disease (AD), may show comparable profiles. This raises the question of whether there is an issue with the establishment of methylation profiles in the pathogenesis of AD. DNA methylation occurs through the transfer of a methyl group to the 5th carbon atom of the cytosine base by DNA methyl transferase enzymes (*DNMT*s) that changes the transcriptional regulation of the genes. Ten-eleven translocation (TET) factors function in the removal of the methyl group from methylated cytosine, in contrast to DNMT enzymes. In this study, we aimed to investigate the roles of DNMT and TET genes responsible for the regulation of the DNA methylation profile in the pathogenesis of AD.

## Materials and methods

After receiving approval from the Erciyes University Human Research Ethics Committee and obtaining consent from the participants, this study included patients diagnosed with AD who presented to the Faculty of Medicine, Department of Dermatology and Venereal Diseases, Erciyes University. In addition, healthy individuals matched for age and sex were also included in the study (Decision No. 2020/416). The blood and skin biopsy samples collected from the patients and healthy participants were sent to the Betül-Ziya Eren Genome and Stem Cell Center, Department of Genome, Erciyes University, for molecular studies.

The recruitment method for the patients included in the study was prospective. Patients aged 2–18 years who presented to the Faculty of Medicine, Department of Dermatology and Venereal Diseases Outpatient Clinic, Erciyes University and diagnosed with AD were included in the study. To be eligible, these patients should not have any chronic diseases other than AD and must not have received topical treatments in the previous 2 weeks or systemic treatments in the previous 4 weeks for the management of AD. The SCORAD scores of the patients were calculated, and 15 patients with scores higher than 25 were included in the study.

The control group consisted of 15 healthy individuals aged 2–18 years, without chronic or dermatological diseases. These participants were scheduled for fracture reduction or arthroscopic knee procedures at the Faculty of Medicine, Department of Orthopedics and Traumatology, Erciyes University.

### Skin biopsy and blood sample collection

In this study, tissue samples were collected from both lesional (affected) and non-lesional (unaffected) skin under standardized conditions. For lesional samples, 15 patients with atopic dermatitis (AD) underwent 3 mm punch biopsies of the skin in the popliteal fossa (the area behind the knee) after receiving local anesthesia. The procedure was conducted under sterile conditions to ensure sample integrity and to prevent infection. For non-lesional samples, 15 healthy volunteers had normal skin biopsies taken from the same anatomical region using the same method. This consistent approach allowed for a direct comparison between the lesional skin of the AD patients and the normal skin of the healthy controls. Also, 3 mm punch biopsies of the skin were taken from the popliteal fossa of 15 healthy volunteers.

Blood samples were collected from the blood vessels into EDTA tubes from both patients and healthy volunteers on the same day as when the tissue samples were obtained. This simultaneous collection ensured consistency in sample timing and minimized potential variability.

These tissue and blood samples were stored at −80°C until the study began, following the completion of RNA isolation.

### Total RNA isolation and real-time PCR

Total RNA was isolated from skin biopsies and blood samples using Trizol (Thermo Fisher Scientific, MA, USA). The concentration and quality of the RNA were determined using a Nanodrop device (Shimadzu, Japan). cDNA was synthesized from the RNA samples using an Evo Script cDNA synthesis kit (Roche, Mannheim, Germany). The cDNA synthesis procedure was conducted according to the manufacturer’s protocol. A total of 3 μL of RNA was added to the reaction mix, resulting in a final concentration of 2.5 μg. Quantitative PCR (q-PCR) was then performed using the high-throughput LightCycler 480 II Real-Time PCR system (Roche, Germany, Mannheim). The cDNA was diluted with nuclease-free water at a ratio of 1:5. Syber Green Master (Roche, Germany, Mannheim, Cat No: 04707516001) was used for detection. The reaction mix was prepared according to the manufacturer’s instructions, with 5 μL of the cDNA added to the mix. The primers used in the study are listed in Supplementary Table 1. The ACTB gene was used as a housekeeping gene. For all samples, each gene was run in duplicate on the same plate. Ct values were normalized using the 2^−ΔΔCt^ method (9, 10).

### Statistical analysis

The conformity of the data to a normal distribution was evaluated using histograms, q–q plots, and the Shapiro–Wilk test, while the homogeneity of variance was evaluated using Levene’s test. Student’s *t*-test was performed to compare the differences in the mRNA expression levels of eight different genes found in the tissue and blood samples collected from the patient and control groups.

The relationship between quantitative data was evaluated using the Spearman correlation analysis. These data were evaluated using GraphPad Prism (version 8.0.1), and a *p*-value of <5% was considered statistically significant.

## Results

A total of 15 patients with AD were included in the study. Among these patients, eight (53.33%) were girls and seven (46.67%) were boys. The control group consisted of 15 healthy individuals, of which 8 (53.33%) were girls and seven (46.67%) were boys. The mean age of the patients was 8.8 years, and the mean age of the healthy individuals was 9.8 years. There was no statistically significant difference in terms of age and sex between the groups (*p* > 0.05). Six (40%) patients had only facial and neck involvement, while four (27%) patients had neck, facial, and flexural involvement. Five (33%) patients had only flexural involvement. Among the 15 patients, 3 (20%) were diagnosed with asthma accompanying atopic dermatitis, 1 (6.66%) had a food allergy (peanut), and 2 (13.33%) were diagnosed with allergic rhinitis. Three (20%) patients had a family history of asthma (mother and/or father), and two (13.33%) had a diagnosis of atopic dermatitis. The serum IgE levels were elevated in nine (60%) participants in the patient group, according to their age range. The mean SCORAD index of the patient group was calculated to be 36.9 (Table 1).

**Table 1:** Demographic characteristics of the patients with AD and the control group.

	Patients with AD	Control group
Sex		
Female	8 (53.33%)	8 (53.33%)
Male	7 (46.67%)	7 (46.67%)
**Mean age (years)**	8.8 (2–16)	9.8 (4–15)
Female	9.75 (2–15)	10.62 (4–16)
Male	7.71 (4–16)	9.12 (5–16)
Clinical presentation		
Flexural involvement	5 (33%)	
Facial and neck involvement	6 (40%)	–
Neck, facial, and flexural involvement	4 (27%)	–
Comorbidities		
Asthma	3 (20%)	–
Food allergy (peanut)	1 (6.66%)	–
Allergic rhinitis	2 (13.33%)	
**Family history of asthma**	3 (20%)	–
**Family history of atopic dermatitis**	2 (13.33%)	–
Serum IgE elevation		
Female	5	–
Male	4	–
Mean SCORAD index		
Female	35.28	–
Male	38.76	–

### Transcript levels of the *DNMT1, DNMT2, DNMT3A, DNMT3B, DNMT3L, TET1, TET2*, and *TET3* genes in the lesional skin and blood samples of AD patients

The mRNA expression levels of eight different genes found in the tissue and venous blood samples obtained from the AD patients and the healthy control group are summarized in Supplementary Table 1.

**Figure 1:**
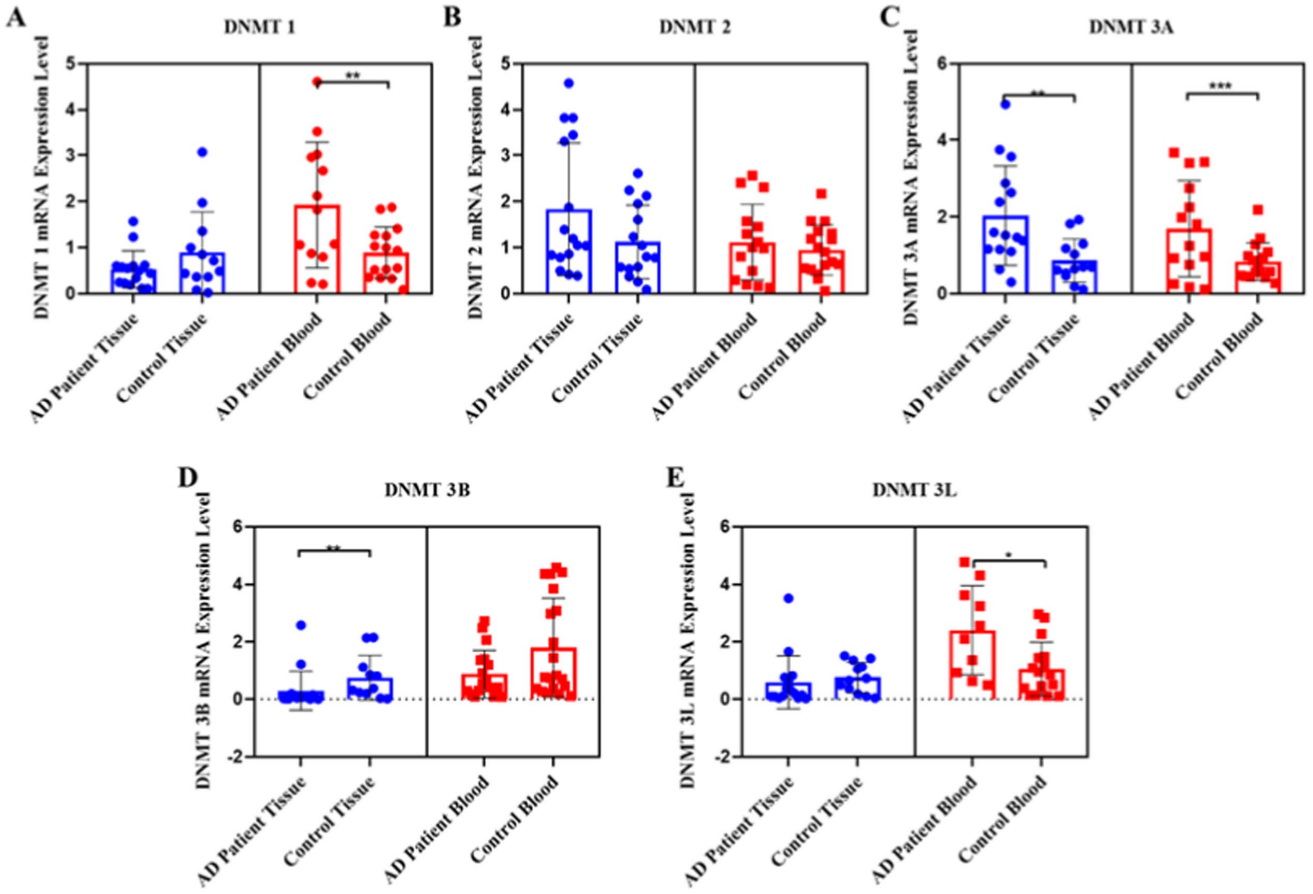
Pooled demonstration of the transcript levels of DNMT1, DNMT2, DNMT3A, DNMT3B, and DNMT3L genes in the blood and tissue samples obtained from AD patients and the healthy control group. Graphs for DNMT1, DNMT2, DNMT3A, DNMT3B, and DNMT3L are shown in panels **(A–E)**, respectively.

When DNMT1 transcript levels in the tissue and blood samples from the AD patients were compared with those from the control group, it was found that the *DNMT1* levels in the tissue samples were similar between AD patients and the control group, with no significant difference. However, the *DNMT1* levels were lower in the tissue samples of AD patients compared to the control samples. In contrast, a significant two-fold increase in the *DNMT1* transcript levels was observed in the blood samples of AD patients compared to the control group (*p* = 0.008) (Figure 1A). Although there was an observed increase in the *DNMT2* transcript levels in both tissue and blood samples from AD patients, the difference was not statistically significant (Figure 1B). The transcript levels of the *DNMT3A* gene were significantly elevated, showing a two-fold increase in both blood (*p* = 0.001) and tissue samples (*p* = 0.001) of AD patients compared to the control group (Figure 1C). In contrast, the *DNMT3B* transcript levels in the tissue samples of AD patients exhibited a four-fold reduction compared to the control group (*p* = 0.0030); however, no significant difference was observed in the blood samples (Figure 1D). The *DNMT3L* gene showed a three-fold increase in the transcript levels exclusively in the blood samples of AD patients compared to the control group (*p* = 0.0231) (Figure 1E).

**Figure 2:**
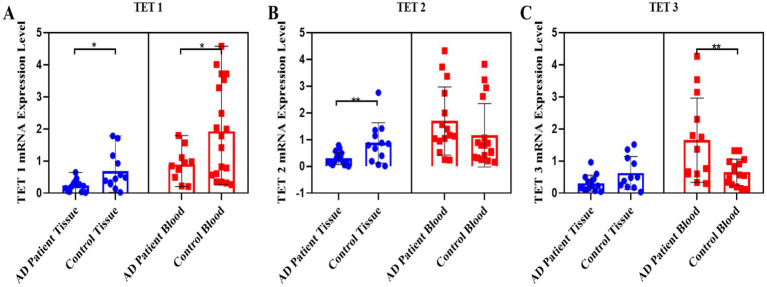
Pooled demonstration of the transcript levels of the TET1, TET2, and TET3 genes in the blood and tissue samples obtained from AD patients and the healthy control group. Graphs for TET1, TET2, and TET3 are shown in panels **(A–C)**, respectively.

Regarding the TET gene family, the *TET1* transcript levels decreased two-fold in both blood (*p* = 0.015) and tissue samples (*p* = 0.013) of AD patients compared to the control group (Figure 2A). A two-fold decrease in the *TET2* transcript levels was specifically observed in the skin tissue samples of AD patients compared to the control group (*p* = 0.008) (Figure 2B). In contrast, the *TET3* transcript levels increased approximately two-fold in the blood samples of AD patients compared to the control group (*p* = 0.0093), with no significant difference observed in the tissue samples (Figure 2C).

In addition, the potential relationship between the SCORAD scores of the patients and the expression levels of *DNMT1, DNMT2, DNMT3A, DNMT3B, DNMT3L, TET1, TET2*, and *TET3* genes in the blood samples and lesional tissues was evaluated using the correlation analysis. However, no significant positive or negative relationship was found.

## Discussion

Atopic dermatitis (AD) is a chronic inflammatory skin disease that affects people globally. This condition not only has a significant impact on the quality of life for those affected but also places a considerable burden on healthcare systems and economies (11). Understanding the prevalence of AD in both developed and developing countries is essential for effective public health strategies and healthcare planning. While higher prevalence rates have been reported in developed countries, developing countries face challenges including limited access to healthcare services in managing AD. The increased prevalence in developed countries can be attributed to better healthcare infrastructure and advanced diagnostic capabilities (12–13). Environmental factors play a significant role in the development and exacerbation of AD. Factors such as air pollution, household dampness, mold, proximity to green spaces, and dietary habits can influence the development of AD. The high prevalence of AD in developing countries is associated with the impact of these environmental and lifestyle factors (14). The complex pathogenesis of AD may be influenced by interactions between genetic predisposition, immune dysregulation, and environmental stress factors (15). In addition, exposure to allergens, especially inhaled allergens such as house dust mites, can increase the incidence of AD (16–18). Stress also has been identified as an important factor in both the development and exacerbation of AD (19).

Epigenetic modifications refer to reversible changes in gene expression without alterations to the DNA sequence and occur at all stages of development or in response to environmental factors (7). Studies have previously shown that AD has a hereditary component and that epigenetic mechanisms play a role in its pathogenesis (20).

There are three main epigenetic mechanisms: DNA methylation at the DNA level, histone modifications affecting histone tails, and small RNAs (smRNAs) and long non-coding RNAs (lncRNAs) at the RNA levels. “Epigenome” refers to the global profile of gene expression, and epigenetic mechanisms can alter the epigenome.

DNA methylation, one of the epigenetic mechanisms, involves the transfer of a methyl group to the 5th carbon atom of cytosine by DNMTs and generally mediates the suppression of gene mRNA expression. When studies in the literature on AD-related DNA methylation were reviewed, it was found that there were changes in the methylation profiles of many genes associated with AD. However, changes in the methylation profiles of different genes alone cannot account for the cause of this disease, just as mutations alone cannot provide a complete explanation (5, 21–23). Our hypothesis suggests that these changes stem from a global shift in the genome’s methylation profile. The literature has yet to clarify how changes in the expression of genes that establish and remove the global DNA methylation profile play a role in the pathogenesis of AD. *DNMT* and *TET* enzymes play a role in the establishment of the methylation profile of the genome. Consequently, changes in the expression of these genes could lead to changes in the methylation profiles of many genes controlled by DNA methylation, which in turn may affect their expression levels (5, 21–23).

Our primary aim in this study was to analyze the transcript profiles of DNMT and TET genes and investigate how these genes contribute to AD pathogenesis. These genes are crucial in regulating global DNA methylation changes in the skin and blood of AD patients. Previous research has examined the methylation profiles of various individual genes, but these studies have not provided a comprehensive view of hyper- or hypo-methylation states in the context of AD. This suggests that focusing solely on the methylation status of individual genes or global methylation changes may not fully elucidate the complex etiology of AD.

We hypothesized that the discrepancy arises because global DNA methylation patterns are influenced by environmental factors, which can lead to alterations in the methylation pattern. DNMT and TET genes that are responsible for maintaining and modifying this global methylation map may therefore play a significant role in the development of AD. Fundamental changes in epigenetic mechanisms often begin with alterations in the transcript levels of these genes. Although changes in transcript levels may not always correspond directly to protein levels, they can indicate shifts in underlying epigenetic regulation. Understanding these changes could provide valuable insights into how epigenetic mechanisms contribute to AD pathogenesis and highlight potential targets for therapeutic intervention.

In our study, we found that the expression levels of *DNMT1* in the peripheral blood samples of AD patients were higher compared to those of the control group. This finding contrasts with a study performed by Nakamura et al. (24), which served as the starting point for our research. This study focused solely on blood samples of AD patients and demonstrated that DNMT1 transcript levels in peripheral blood mononuclear cells were significantly lower in AD patients compared to the control group (24).

When the literature was reviewed, various studies showed changes in the DNA methylation profile of different genes, generally in the promoter regions, in AD patients. However, the relationship between changes in the expression levels of genes involved in DNA methylation and the pathogenesis of AD has not yet been studied. *DNMT1* is a general DNA methyltransferase that, after establishing DNA methylation patterns, transfers methylation to the next generation by copying the methylation onto the newly formed strand based on the old DNA strand. *DNMT3A and DNMT3B* are responsible for establishing new DNA methylation patterns through *de novo* DNA methylation activities by methylating unmethylated CpG dinucleotides during early embryonic stages and germ cell differentiation (25, 26).

In our study, we found that the *DNMT1* transcript level increased two-fold in the blood samples of AD patients compared to the control group. *DNMT1* is generally responsible for maintaining DNA methylation, and its increase in blood may indicate a shift toward hypermethylation in the genome of AD patients. *DNMT3A* and *DNMT3B* are responsible for *de novo* methylation profile changes after mitosis or in response to environmental factors. It was found that the *DNMT3A* transcript level increased two-fold both in the skin and blood samples of AD patients, whereas the transcript level of DNMT3B decreased four-fold in both skin tissue and blood samples of AD patients compared to the control group. There is limited information in the literature regarding how *DNMT3A* and *DNMT3B* genes establish a de novo methylation pattern. One study suggested that *DNMT3A* and *DNMT3B* function differently. It was shown that an increased *DNMT3A* transcript level is associated with triggering cellular aging, while a decreased *DNMT3B* transcript level indicates increased DNA damage and disruption of DNA repair mechanisms (27). In light of these data available in the literature, our results indicate that cellular aging may be triggered by increased expression of the *DNMT3A* gene in AD patients. Additionally, the decrease in *DNMT3B* expression could indicate the presence of increased DNA damage and a possible disruption in the repair mechanisms of the damage and the DNA methylation pattern is changed. However, other studies, especially those that assess the protein levels, are required to confirm these data completely. When the data we obtained were evaluated alongside the findings in the literature, the main point we aimed to highlight in this study was that changes in epigenetic mechanisms in AD patients are influenced by environmental factors (28), and these altered epigenetic mechanisms affect the transcript levels of genes that establish the global DNA methylation map. As a result, *DNMTs* and *TETs*, whose transcript levels change, may affect the global methylation map from the outset.

The *DNMT2* enzyme is an RNA methyltransferase that plays a crucial role in tRNA and mRNA methylation. It has been shown that the *DNMT2* enzyme is essential for the recognition of DNA damage, DNA recombination, and mutation repair. Furthermore, global DNA methylation cannot occur in embryonic stem cells in the absence of this enzyme (29, 30). In our study, no significant differences in the DNMT2 levels in blood or skin tissue samples were observed between AD patients and the control group. *DNMT1* and *DNMT2* perform similar roles on different nucleic acids. *DNMT1* is directly involved in DNA methylation, whereas *DNMT2* is more closely associated with methylating tRNA and other small RNA molecules and does not directly impact DNA methylation. In our study, while *DNMT1* exhibited high expression levels in the blood of AD patients, no significant differences were observed in *DNMT2*. This lack of differentiation may suggest that DNA methylation, rather than RNA methylation, plays a more significant role in AD pathogenesis. To confirm this hypothesis, more detailed methylation screenings should be conducted to provide further insights. In our study, we measured the transcript levels of the *TET* genes, which are responsible for the removal of DNA methylation, in the skin tissue and blood samples of AD patients and compared them those of healthy controls. The *TET1* transcript levels were detected to be decreased two-fold in the blood and skin tissue of AD patients compared to the control group. The *TET2* transcript levels were also decreased two-fold in the skin tissue of AD patients compared to the control group, although the difference was not significant; however, they were increased in the blood samples. It was shown in the literature that a decrease in *TET2* expression led to reduced potential for Treg cell activation (31). The *TET3* transcript levels were found to be increased by approximately two-fold in the blood of AD patients.

There are no other studies in the literature that have evaluated the roles of *DNMT* and *TET* transcript levels in the pathogenesis of AD. However, the roles of *DNMTs* and *TETs* transcript levels in disease pathogenesis have been shown in other conditions, particularly in the context of the atopic march (32–41).

It was demonstrated in a study that particulate matter (PM) accelerates the aging process in human keratinocytes and mouse skin tissue by affecting epigenetic inheritance (42). This finding supports the data that the altered *DNMT3A* and *3B* transcript profiles in AD patients accelerate the aging process.

It was also demonstrated that cells treated with PM 2.5 show cellular aging characteristics, leading to a decrease in *DNMT1* and *DNMT3B* expression levels and an increase in *TET1* transcript levels, and the genome shifts to a hypomethylated state with allergen exposure (42).

When we compared our data with the findings in the literature, we observed that changes in the transcript levels of *DNMT* and *TET* genes, especially *DNMT3A* and *DNMT3B*, could trigger DNA damage and accelerate cellular aging (26). However, we believe that epigenetic destabilization plays an important role in the onset of AD, and our main point in the study is that changes in the transcript levels of *DNMT* and *TET* genes can change the global methylation map as well.

The increase in *DNMT3A* expression levels observed in the lesional tissues of the patients with AD and the elevated levels of *DNMT1, DNMT3A, DNMT3L*, and *TET3* in the blood, compared to the controls, may be attributed to cellular stress resulting from inflammation associated with AD. This inflammatory process could potentially trigger epigenetic modifications, thereby inducing changes in various transcription factors and modulating the expression of genes encoding DNMT and TET enzymes. To better understand these findings, it would be beneficial to assess the expression levels of these genes in non-lesional biopsy samples, which would provide insights into the effects of the inflammatory process. This represents a significant limitation of our study.

Consistent with the data found in the literature, the totality of our available data indicates that changes in DNMT and TET transcript levels, especially DNMT3A and DNMT3B, trigger DNA damage, accelerate cellular aging, and lead to *de novo* methylation profile changes in AD. According to the results we obtained, we believe that epigenetic destabilization plays a role in the onset of AD. The changes in DNMT and TET transcript levels in AD patients were significantly higher than in the control group. Our findings indicate that in AD disease both the methylation profile of individual genes and the global genome methylation pattern are altered. Changes in the global DNA methylation pattern can activate some genes and repress others. In other words, it alters the epigenome and may lead to genomic instability. The data we obtained are preliminary and indicate that the DNA methylation pattern changes in AD, especially under the influence of skin tissue. These findings may contribute to the diagnosis of AD patients and the development of new treatment strategies. This should be supported by future studies involving comprehensive transcriptome and protein analyses. The key point at this stage is that revealing the genome’s tendency toward a hypermethylated or hypomethylated state can provide opportunities for either negative or positive modulation of methyl group donor pathways, which may help in controlling AD symptoms.

## Data Availability

The raw data supporting the conclusions of this article will be made available by the authors, without undue reservation.
