# Supplementary material for: Regulation of DNA methylation in lesional tissue of children with atopic dermatitis
Source: Front Med (Lausanne). 2025 Mar 3;12:1531777. doi: 10.3389/fmed.2025.1531777 (PMC11913172; doi:10.3389/fmed.2025.1531777)
Supplement: Supplementary file 1 [file Table_1.docx]

**Supplemental Table 1**. Primer sequences of DNMT1, DNMT2, DNMT3A, DNMT3B, DNMT3L, TET1, TET2 and TET3 genes

| Genes | Primary Sequence **(5'>3')** | The Size of the Product |
| --- | --- | --- |
| *DNMT1-F* | AGAACGGTGCTCATGCTTACA | 171 Bp |
| *DNMT1-R* | CTCTACGGGCTTCACTTCTTG |  |
| *DNMT2-F* | TGCCAAGACGATTGAAGGCAT | 180 Bp |
| *DNMT2-R* | GCAGGGAGGGCTCATTAAAAT |  |
| *DNMT3A-F* | CACACAGAAGCATATCCAGGAGTG | 551 Bp |
| *DNMT3A-R* | AGTGGACTGGGAAACCAAATA |  |
| *DNMT3B-F* | ACCTCGTGTGGGGAAAGATCA | 192 Bp |
| *DNMT3B-R* | CCATCGCCAAACCACTGGA |  |
| *DNMT3L-F* | AAGTTCCTGGATGCCCTCTT | 495 Bp |
| *DNMT3L-R* | GCCGTACACAAGATCGAAGG |  |
| *TET1-F* | CGCTACGAAGCACCTCTCTTA | 189 Bp |
| *TET1-R* | CTTGCATTGGAACCGAATCATTT |  |
| *TET2-F* | ATACCCTGTATGAAGGGAAGCC | 95 Bp |
| *TET2-R* | CTTACCCCGAAGTTACGTCTTTC |  |
| *TET3-F* | TCCAGCAACTCCTAGAACTGAG | 216 Bp |
| *TET3-R* | AGGCCGCTTGAATACTGACTG |  |
| *ACTB-F* | GCCGTCTTCCCCTCCA | 87 Bp |
| *ACTB-R* | CTCGTCGCCCACATAGGAA |  |
